# Predicting response to anti-TNFα therapy among patients with axial spondyloarthritis (axSpA): results from BSRBR-AS

**DOI:** 10.1093/rheumatology/kez657

**Published:** 2020-01-28

**Authors:** Gary J Macfarlane, Ejaz Pathan, Gareth T Jones, Linda E Dean

**Affiliations:** 1 Epidemiology Group, School of Medicine, Medical Science and Nutrition, University of Aberdeen, Aberdeen, UK; 2 Spondylitis Program, Department of Rheumatology, Toronto Western Hospital, Toronto, Canada

**Keywords:** axial spondyloarthritis, ankylosing spondylitis, response, treatment, anti-TNF-alpha, biologic therapy

## Abstract

**Objectives:**

While many axSpA patients, eligible to receive anti-TNFα therapy, derive benefit when prescribed them, some patients do not. The current study aims to identify modifiable targets to improve outcome as well as non-modifiable targets that identify groups less likely to derive benefit.

**Methods:**

The BSRBR-AS is a prospective cohort study of axSpA patients who, at recruitment, were naïve to biologic therapy. Those in the ‘biologic’ sub-cohort commenced their first anti-TNFα therapy at recruitment or during follow-up. Prior to commencement, information was collected on socio-economic, clinical and patient-reported factors. Outcome was assessed according to ASAS20, ASAS40, ASDAS reduction and achieving a moderate/inactive ASDAS disease state.

**Results:**

335 participants commenced their first anti-TNFα therapy and were followed up at a median of 14 (inter-quartile range 12–17) weeks. Response varied between 33% and 52% according to criteria used. Adverse socio-economic factors, fewer years in education predicted lower likelihood of response across outcome measures as did not working full-time. Co-morbidities and poor mental health were clinical and patient-reported factors, respectively, associated with lack of response. The models, particularly those using ASDAS, were good at predicting those who did not respond (negative predictive value (NPV) 77%).

**Conclusion:**

Some factors predicting non-response (such as mental health) are modifiable but many (such as social/economic factors) are not modifiable in clinic. They do, however, identify patients who are unlikely to benefit from biologic therapy alone. Priority should focus on how these patients receive the benefits that many derive from such therapies.

**Rheumatology key messages**

- Amongst axSpA patients receiving their first anti-TNFα therapy, 33–52% respond by first follow-up visit.
- Modifiable predictors of anti-TNFα non-response in axSpA include mental health but not consistently disease activity.
- Non-modifiable predictors of anti-TNFα non-response identify axSpA patients who may benefit from additional support.

## Introduction

The introduction of anti-TNF-α therapies has transformed the outlook for patients with inflammatory arthritis, including axial spondyloarthritis (axSpA). They have resulted in clinically important benefits in terms of improving function and quality of life [1, 2] and have improved wider outcomes such as fatigue (in RA) and work productivity (in AxSpA) [3, 4]. Of course, the studies report average affects and within approaches to management that show overall benefit, there will be people who do not respond to therapy or show less improvement. Indeed, in a real-world setting, of patients with axSpA commenced on a first anti-TNFα agent, around one in four will no longer be on the agent 12 months later [5].

Patients who are obese have been reported as less likely to respond to anti-TNFα therapies in axSpA and RA [6] and RA patients with symptoms of depression are less likely to achieve a good response to biologic therapy [7]. It has been hypothesized that patients with axSpA who have co-morbid fibromyalgia may have distorted disease indices, receive anti-TNFα therapy inappropriately and if they do, that they derive less benefit. However we have shown, using data from the British Society for Rheumatology Biologics Register in Ankylosing Spondylitis (BSRBR-AS) that there is only a very small effect of meeting criteria for fibromyalgia *per se* on disease indices such as the Bath Index of Disease Activity (BASDAI) and the magnitude of improvement is no different to those who do not meet criteria for fibromyalgia [8].

The aim of the current study was to identify factors (including socio-economic, clinical and patient reported) that characterized axSpA patients who were less likely to respond to their first anti-TNFα therapy. Identifying such factors is, in general, important in terms of providing optimal management and can provide a focus of research to understand the mechanisms that lead to lack of improvement in people with certain characteristics.

## Methods

The BSRBR-AS is a prospective cohort study of axSpA patients who, at recruitment, were naïve to biologic therapy. Recruitment took place in 83 secondary care centres across the Great Britain between December 2012 and December 2017, for those patients aged at least 16 years meeting the Assessment of SpondyloArthritis international Society (ASAS) imaging criteria for axSpA [9] or the modified New York (mNY) definition of ankylosing spondylitis (AS) [10]. From November 2014, those meeting the ASAS clinical criteria were also eligible. Details of the study protocol have previously been published [11]. There are two sub-cohorts: those commencing their first anti-TNFα therapy at the time of recruitment (primarily the agents adalimumab, etanercept and certolizumab pegol) thereafter named the ‘biologic cohort’ and those remaining on other therapies (‘non-biologic cohort’). The biologic cohort was followed-up at 3 months and 6 months, and both cohorts were seen at 12 months and yearly thereafter to a maximum of 5 years. In addition to clinical data, patient reported questionnaires were completed at each follow-up. If a patient in the non-biologic cohort commenced anti-TNFα therapy, they switched sub-cohort and began a new follow-up schedule.

The primary outcome of interest for the current analysis is response to first anti-TNFα therapy at initial follow-up, defined as the first contact with the study in the period 10 weeks to 9 months after commencement. This period was chosen in order to measure outcome within the first two follow-up periods of the study (but allowing for early or late clinic visits). We looked at a variety of outcome measures to determine to what extent there was consistency in predictors or alternatively whether predictors were importantly related to the precise outcome measure used. Response was therefore defined in the following ways:

- meeting ASAS20 and ASAS40 improvement criteria [12, 13];
- exhibiting a clinically important improvement in the Ankylosing Spondylitis Disease Activity Score (ASDAS) – a reduction of ≥1.1; and
- moving from a high or very high ASDAS disease activity state (score ≥2.1) to a moderate or inactive disease state (score <2.1) [14, 15].

Measures collected at recruitment (baseline), used in the current analysis as potential predictors of response include those listed below.

### Clinical data

The following were recorded: the classification criteria fulfilled (ASAS imaging, ASAS clinical or mNY), presence of extra-spinal manifestations (history of uveitis, psoriasis, IBD, peripheral joint involvement and clinically assessed heel enthesitis and dactylitis), count of comorbidities (specifically, the presence of angina, congestive heart failure, stroke, hypertension, diabetes, asthma, bronchitis, peptic ulcer, liver disease, renal disease, tuberculosis, demyelination, depression and malignancy). The following were measured: BMI, inflammatory markers (CRP or ESR), HLA-B27 status, physician-assessed swollen/tender joint count and the BASMI scored 0 (least) to 10 (most severe) [16].

### Patient reported socio-economic, health and lifestyle measures

Using study questionnaires, information was collected on: socio-economic factors (level of education, employment status at recruitment), lifestyle factors (tobacco smoking and alcohol intake) and quality of life, assessed by the AS Quality of Life index (ASQoL) scored from 0 (best) to 18 (worst) [17], and the Short Form 12 Physical and Mental component scores, scored from 0 (worst) to 100 (best) [18]. Mental health was assessed by the Hospital Anxiety and Depression Scale (HADS) scored from 0 (best) to 21 (worst) [19] and overall work and other activity impairment using the Work Productivity and Activity Impairment Specific Health Problem (WPAI: SHP), both scored from 0–100% [20, 21]. Spinal pain was assessed using a 10 cm visual analogue scale, fatigue through the Chalder Fatigue Scale (CFS) (0–11) [22], and sleep disturbance by the Jenkins Sleep Evaluation Questionnaire (0–20) [23], with higher scores on each indicating worse state. Lastly, the Bath Ankylosing Spondylitis indices were included to provide measures of disease activity (BASDAI), function (BASFI) and global health (BAS-G) [all scored from 0 (best) to 10 (worst)] [24–26]. Information provided by participants on their address was used to derive a measure of local area deprivation according to quintiles (based on the distribution of their country of residence within the UK) from 1 (least deprived) to 5 (most deprived) [27, 28]. For those recruited after September 2015, information was collected on the 2011 research criteria for fibromyalgia [29].

The BSRBR-AS received ethical approval from the UK National Research Ethics Service (NRES) Committee North East – County Durham and Tees Valley (REC ref 11/NE/0374).

### Statistical analysis

For the purpose of the current analysis, participants who joined the biologic sub-cohort were eligible. The relationship between the clinical and patient reported (including socio-economic) baseline factors and each of the follow-up response criteria were assessed, initially by logistic regression models, with results given as odds ratios (OR) and 95% CI. Continuous variables or counts were retained as such during all analyses and assessed for their association with outcome per unit increase. For dichotomous factors, such as a history of uveitis, the presence of each was assessed for association with outcome compared with the absence (yes *vs* no). Smoking status was categorized into never, ex and current smoking. Current smokers were further dichotomized as </≥ the median level of smoking (10 products/day). Alcohol consumption was categorized as never, ex and current, with current drinkers dichotomized ≤/>14 units of alcohol per week (i.e. the maximum consumption recommended by the National Health Service [30]). For all multi-level categorical factors, including smoking and alcohol status, reference categories were selected.

Those factors reaching a significance threshold of *P *≤0.2 were offered to individual forward stepwise logistic regression models in order to determine the group of factors that best predict response (according to each of the response criteria examined). Factors entered the stepwise model at *P *≤ 0.15 and exited at *P *≥ 0.10. The fit of the final models was assessed through the calculation of the area under the receiver operator characteristic (ROC) curve (95% CI), sensitivity and specificity in addition to the positive and negative predictive values (PPV/NPV).

All analysis was conducted using STATA (StataCorp LP version 15.0) and uses the August 2017 version of the dataset.

## Results

The timing of patient follow-up from commencing first anti-TNF therapy to measuring response in the current analysis is a median of 14 weeks with an inter-quartile range (IQR) of 12–17 weeks. At this time, 95% were still taking their first anti-TNF.

### Baseline characteristics of study population

A total of 335 participants were eligible for the current analysis: 69% male, with median age 47 years (IQR 36–56) and median BMI of 27.2kgm^−2^ (IQR 24.1, 31.1) (Tables 1 and 2). The only important difference between those who were included in this analysis (i.e. had follow-up clinic visit and provided patient reported outcome measures) and those who were not, was deprivation. Of those included, 49.8% were from the two least deprived quintiles in comparison to 34.4% of those not included. The majority of participants (63.9%) were, at time of commencing therapy working full or part-time while 19.2% were unemployed or had retired due to ill-health. Approximately two-thirds of participants met mNY criteria for AS (61.2%), one-third met ASAS imaging criteria but not mNY criteria for AS (34.9%), while only a small proportion met only ASAS clinical criteria (3.9%), mainly as a result of these only become eligible criteria part-way through the recruitment period; 76.5% of those tested were HLA B-27 positive. In terms of disease activity, 95.7% were classified as having high or very high disease activity with an ASDAS score ≥2.1, while 90.8% had a BASDAI score of at least 4. Overall, the patient population had high median levels of fatigue (CFS score 6.0), sleep disturbance (Jenkins score 14.0), anxiety (HADS anxiety score 9.0) and depression (HADS depression score 8.0) (Table 2).

**Table 1:** Baseline socio-economic and lifestyle characteristics of those commencing biologic therapy

		*n*	Median (IQR)
Age	years	335	46.6 (36.4, 56.1)
		*n*	%
Gender	Male	230	(68.7)
Education	Secondary school	117	(35.4)
	Apprentice	36	(10.9)
	College	86	(26.0)
	University degree	67	(20.2)
	Further degree	25	(7.5)
Employment	Full-time	165	(49.6)
	Part-time	48	(14.4)
	Unpaid/seeking	15	(4.5)
	Retired	37	(11.1)
	Retired/unemployed due to ill-health	64	(19.2)
	Student	4	(1.2)
Deprivation^a^	1 (least deprived)	80	(23.9)
	2	87	(26.0)
	3	66	(19.7)
	4	55	(16.4)
	5 (most deprived)	47	(14.0)
Smoking	Never	142	(43.3)
	Ex-smoker	111	(33.8)
	Current – light	31	(9.5)
	Current – heavy	44	(13.4)
Alcohol drinking	Never	26	(7.8)
	Ex drinker	57	(17.1)
	Current – ≤14 units/week	228	(68.5)
	Current – >14 units/week	22	(6.6)

IQR, inter-quartile range.

a Quintiles of general population distribution.

**Table 2:** Baseline clinical and patient*-*reported health characteristics of those commencing biologic therapy

		*n*	%
Disease criteria	Modified New York	205	(61.2)
	ASAS imaging	117	(34.9)
	ASAS clinical	13	(3.9)
First biologic	Adalimumab	223	(66.6)
	Etanercept	78	(23.3)
	Certolizumab pegol	33	(9.8)
	Golimumab	1	(0.3)
Extra-spinal manifestations	Heel enthesitis present	38	(11.4)
	Uveitis present	78	(23.4)
	Dactylitis present	16	(4.8)
	Psoriasis present	32	(9.6)
	IBD present	38	(11.4)
	Peripheral joint disease present	74	(22.2)

		*n*	Median (IQR)

BMI	kg/m^2^	267	27.2 (24.1, 31.1)
CRP (mg/dl)	mg/dl	287	0.70 (0.2, 2.2)
Tender joint count	range: 0–44	323	0 (0, 0)
Swollen joint count	range: 0–40	318	0 (0, 0)
Spinal mobility	BASMI: 0 (best) – 10 (worst)	259	4.4 (2.8, 5.6)
Comorbidity count	range: 0 – 14	332	0 (0, 1)
SF-12 – MCS	Scored: 0 (worst) – 100 (best)	326	42.8 (35.5, 53.1)
SF-12 – PCS	Scored: 0 (worst) – 100 (best)	326	32.3 (24.1, 39.8)
Disease activity	BASDAI: 0 (best) – 10 (worst)	335	6.7 (5.4, 7.8)
	ASDAS: (higher score worse)	300	3.7 (3.2, 4.5)
Physical function	BASFI: 0 (best) – 10 (worst)	335	6.7 (5.0, 8.1)
Global health	BASG: 0 (best) – 10 (worst)	334	7.5 (6.0, 8.5)
Spinal mobility	BASMI: 0 (best) – 10 (worst)	259	4.4 (2.8, 5.6)
Quality of life	ASQoL: 0 (best) – 18 (worst)	332	12 (9, 15)
Fatigue	CFS: 0 (best) – 11 (worst)	335	6 (3, 9)
Sleep disturbance	Jenkins: 0 (best) – 20 (worst)	332	14 (8, 18)
Overall work impairment	%	183	40 (30, 70)
Activity impairment	%	329	70 (50, 80)
Anxiety	HADS: 0 (best) – 21 (worst)	333	9 (6, 12)
Depression	HADS: 0 (best) – 21 (worst)	333	8 (4, 10)
Spinal pain	VAS: 0 (best) – 10 (worst)	335	7 (5, 8)

ASAS: assessment in ankylosing spondylitis; SF-12 MCS: short form 12 mental component score; SF-12 PCS: short form 12 physical component score; BASDAI: Bath ankylosing spondylitis disease activity index; BASFI: Bath ankylosing spondylitis functional index; ASDAS: ankylosing spondylitis disease activity score; BASG: Bath ankylosing spondylitis global score; BASMI: Bath ankylosing spondylitis metrology index; ASQoL: ankylosing spondylitis quality of life index; CFS: Chalder fatigue scale; HADS: hospital anxiety and depression scale; VAS: visual analogue scale; IQR, inter-quartile range.

### Predictors of meeting ASAS20 response

ASAS20 response criteria was achieved by 52% (*n* = 175) of participants. Univariable logistic regression identified several baseline factors associated with lack of response which were eligible as candidates for the stepwise model (*P *≤ 0.2) (Tables 3–5). Demographic factors included: older age, education up to secondary school and not being in full-time employment. Clinical factors included: higher BMI, peripheral joint involvement, no history of uveitis or dactylitis and less favourable BASMI. Patient reported factors included: better disease activity (ASDAS), poorer mental health (Short Form 12 Mental Component Score (SF-12 MCS), HADS), poorer physical function and overall physical health (BASFI, Short Form 12 Physical Component Score (SF12 PCS)), worse quality of life (ASQoL) and higher levels of fatigue and activity impairment. On stepwise logistic regression modelling, three factors independently predicted lack of ASAS20 response (Table 6): not being in full-time employment, higher BMI [OR of response 0.96 per unit increase 95% CI (0.91, 1.003)] and higher initial levels of anxiety [0.94 per unit score increase (0.88, 0.998)]. The model demonstrated a good level of fit (ROC 0.68) with PPV 63%, and NPV 65%.

**Table 3:** Associations of socio-economic baseline factors with each response measure at follow-up (univariable logistic regression analyses)

		ASAS 20 response		ASAS 40 response		ASDAS ≥1.1 reduction		ASDAS score <2.1	
Baseline variables		OR	(95% CI)	OR	(95% CI)	OR	(95% CI)	OR	(95% CI)
Age	Per year	*0.99*	*(0.97, 1.004)*	*0.99*	*(0.97, 1.01)*	0.99	(0.97, 1.01)	0.99	(0.97, 1.01)
Gender	Female *vs* male	1.01	(0.64, 1.60)	*0.69*	*(0.42, 1.14)*	*0.65*	*(0.38, 1.11)*	*0.66*	*(0.37, 1.18)*
Education	Secondary school	*Ref*		*Ref*		*Ref*		*Ref*	
	Apprentice	*1.90*	*(0.89, 4.07)*	*2.02*	*(0.91, 4.47)*	*1.65*	*(0.70, 3.93)*	*1.97*	*(0.80, 4.86)*
	College	*1.00*	*(0.57, 1.75)*	*1.45*	*(0.78, 2.71)*	*0.64*	*(0.33, 1.02)*	*1.17*	*(0.57, 2.42)*
	University degree	*2.31*	*(1.24, 4.30)*	*3.09*	*(1.63, 5.85)*	*1.20*	*(0.91, 2.37)*	*2.64*	*(1.26, 5.53)*
	Further degree	*1.81*	*(0.75, 4.36)*	*1.50*	*(0.58, 3.84)*	*0.53*	*(0.20, 1.45)*	*3.51*	*(1.29, 9.54)*
Employment	Full-time	*Ref*		*Ref*		*Ref*		*Ref*	
	Part-time	*0.48*	*(0.25, 0.92)*	*0.36*	*(0.17, 0.77)*	*0.39*	*(0.18, 0.82)*	*0.23*	*(0.09, 0.57)*
	Unpaid/seeking	*1.24*	*(0.40, 3.78)*	*0.90*	*(0.31, 2.66)*	*0.26*	*(0.07, 1.003)*	*0.21*	*(0.04, 1.04)*
	Retired	*0.73*	*(0.35, 1.49)*	*0.93*	*(0.45, 1.91)*	*0.96*	*(0.43, 2.15)*	*0.68*	*(0.30, 1.55)*
	Retired/ unemployed due to ill-health	*0.26*	*(0.14, 0.49)*	*0.19*	*(0.09, 0.43)*	*0.32*	*(0.16, 0.65)*	*0.04*	*(0.01, 0.18)*
	Student	*1.85*	*(0.19, 18.20)*	*1.36*	*(0.19, 9.87)*	*2.33*	*(0.24, 23.04)*	*2.90*	*(0.29, 28.69)*
Deprivation	1 (least deprived)	*Ref*		*Ref*		*Ref*		*Ref*	
(quintiles)	2	0.96	(0.52, 1.76)	0.71	(0.38, 1.33)	*0.90*	*(0.45, 1.79)*	*0.59*	*(0.29, 1.23)*
	3	0.73	(0.38, 1.41)	0.55	(0.27, 1.09)	*0.49*	*(0.23, 1.03)*	*0.39*	*(0.17, 0.85)*
	4	0.70	(0.35, 1.39)	0.61	(0.29, 1.25)	*0.45*	*(0.21, 0.99)*	*0.43*	*(0.19, 0.99)*
	5 (most deprived)	0.81	(0.39, 1.67)	0.41	(0.18, 0.93)	*0.53*	*(0.22, 1.25)*	*0.36*	*(0.14, 0.93)*

*Italics* indicate variable eligible for stepwise model (*P *<0.2).

ASAS: assessment in ankylosing spondylitis; ASDAS: ankylosing spondylitis disease activity score; OR: odds ratio; ref: reference category.

**Table 4:** Associations of clinical baseline factors with each response measure at follow-up (univariable logistic regression analyses)

		ASAS 20 response		ASAS 40 response		ASDAS ≥1.1 reduction		ASDAS score <2.1	
Baseline variables		OR	(95% CI)	OR	(95% CI)	OR	(95% CI)	OR	(95% CI)
Disease criteria	mNY	*Ref*		*Ref*		*Ref*		*Ref*	
	ASAS imaging	1.05	(0.67, 1.66)	1.16	(0.72, 1.88)	1.11	(0.66, 1.86)	0.89	(0.50, 1.56)
	ASAS clinical	1.09	(0.35, 3.35)	1.35	(0.42, 4.27)	1.19	(0.33, 4.26)	1.84	(0.51, 6.62)
Heel enthesitis	Yes *vs* no	1.29	(0.65, 2.56)	1.37	(0.69, 2.75)	*2.69*	*(1.17, 6.20)*	1.18	(0.51, 2.73)
Uveitis	Yes *vs* no	*1.53*	*(0.91, 2.56)*	1.37	(0.81, 2.32)	*1.49*	*(0.83, 2.67)*	1.15	(0.62, 2.12)
Dactylitis	Yes *vs* no	*2.08*	*(0.71, 6.12)*	1.62	(0.59, 4.46)	*2.15*	*(0.70, 6.61)*	1.63	(0.53, 5.01)
Psoriasis	Yes *vs* no	1.20	(0.57, 2.49)	0.91	(0.42, 2.003)	0.69	(0.30, 1.59)	0.80	(0.33, 1.93)
IBD	Yes *vs* no	0.80	(0.41, 1.58)	*0.60*	*(0.27, 1.31)*	*0.42*	*(0.18, 0.99)*	0.76	(0.32, 1.81)
Peripheral joint disease	Yes *vs* no	*0.63*	*(0.37, 1.06)*	*0.64*	*(0.36, 1.14)*	0.83	(0.46, 1.05)	0.87	(0.46, 1.67)
CRP (mg/dl)	Per unit increase	0.99	(0.99, 1.01)	1.002	(0.99, 1.01)	1.004	(0.99, 1.02)	0.99	(0.97, 1.01)
Tender joint count	Per unit increase	0.97	(0.92, 1.02)	0.98	(0.93, 1.04)	1.003	(0.95, 1.06)	0.98	(0.92, 1.05)
Swollen joint count	Per unit increase	0.96	(0.87, 1.06)	0.94	(0.82, 1.08)	0.97	(0.88, 1.08)	0.93	(0.79, 1.08)
BMI (per kg/m^2^)	Per unit increase	*0.95*	*(0.91, 0.99)*	*0.95*	*(0.90, 0.99)*	0.97	(0.92, 1.02)	*0.94*	*(0.89, 1.001)*
BASMI	Per unit increase	*0.80*	*(0.70, 0.92)*	*0.85*	*(0.73, 0.99)*	0.92	(0.80, 1.07)	*0.83*	*(0.70, 0.99)*
Comorbidity count	Per unit increase	*0.65*	*(0.50, 0.84)*	*0.57*	*(0.42, 0.79)*	*0.60*	*(0.44, 0.82)*	*0.51*	*(0.35, 0.75)*

*Italics* indicate variable eligible for stepwise model (*P *<0.2).

OR: odds ratio; ref: reference category; ASAS: assessment in ankylosing spondylitis; ASDAS: ankylosing spondylitis disease activity score; BASMI: Bath ankylosing spondylitis metrology index.

**Table 5:** Association of patient-reported health and lifestyle factors at baseline, with response at follow-up (multiple univariable logistic regression)

		ASAS 20 response		ASAS 40 response		ASDAS ≥1.1 reduction		ASDAS score <2.1	
Baseline variables		OR	(95% CI)	OR	(95% CI)	OR	(95% CI)	OR	(95% CI)
Smoking	Never	Refer		Ref		Ref		*Ref*	
	Ex	0.82	(0.50, 1.35)	0.83	(0.49, 1.40)	1.01	(0.57, 1.78)	*0.66*	*(0.36, 1.21)*
	Current – light	0.64	(0.29, 1.39)	0.42	(0.16, 1.08)	1.87	(0.74, 4.76)	*0.30*	*(0.10, 0.97)*
	Current – heavy	0.71	(0.36, 1.39)	0.90	(0.44, 1.82)	1.37	(0.63, 2.95)	*0.72*	*(0.32, 1.59)*
Alcohol drinking	Current – ≤14 units/week	*Ref*		*Ref*		*Ref*		*Ref*	
	Never	*0.34*	*(0.14, 0.82)*	*0.22*	*(0.06, 0.74)*	*0.89*	*(0.35, 2.29)*	*0.20*	*(0.04, 0.92)*
	Ex	*0.48*	*(0.27, 0.87)*	*0.39*	*(0.19, 0.80)*	*0.41*	*(0.20, 0.84)*	*0.45*	*(0.21, 0.96)*
	Current – >14 units/week	*1.64*	*(0.65, 4.19)*	*1.38*	*(0.57, 3.32)*	*1.27*	*(0.45, 3.56)*	*1.30*	*(0.42, 4.05)*
SF-12 – MCS	Per unit increase	*1.03*	*(1.01, 1.05)*	*1.02*	*(1.003, 1.05)*	*1.02*	*(1.001, 1.05)*	*1.06*	*(1.04, 1.09)*
SF-12 – PCS	Per unit increase	*1.03*	*(1.01, 1.05)*	*1.03*	*(1.01, 1.06)*	1.01	(0.99, 1.04)	*1.07*	*(1.03, 1.10)*
BASDAI	Per unit increase	0.93	(0.82, 1.05)	0.98	(0.87, 1.11)	*1.16*	*(1.01, 1.33)*	*0.71*	*(0.60, 0.85)*
ASDAS	Per unit increase	*1.18*	*(0.94, 1.48)*	*1.40*	*(1.10, 1.80)*	*2.81*	*(2.03, 3.88)*	*0.74*	*(0.55, 0.99)*
BASFI	Per unit increase	*0.90*	*(0.81, 0.99)*	*0.92*	*(0.83, 1.02)*	0.99	(0.90, 1.11)	*0.70*	*(0.61, 0.81)*
BASG	Per unit increase	0.93	(0.81, 1.05)	0.96	(0.84, 1.10)	0.98	(0.85, 1.13)	*0.68*	*(0.57, 0.81)*
ASQoL	Per unit increase	*0.93*	*(0.88, 0.98)*	*0.95*	*(0.90, 0.99)*	0.98	(0.93, 1.03)	*0.82*	*(0.76, 0.88)*
Fatigue	Per unit increase	*0.94*	*(0.89, 1.002)*	0.97	(0.91, 1.04)	*0.94*	*(0.88, 1.01)*	*0.87*	*(0.80, 0.93)*
Sleep disturbance	Per unit increase	1.01	(0.97, 1.05)	1.01	(0.97, 1.05)	0.99	(0.96, 1.04)	*0.94*	*(0.89, 0.98)*
Activity impairment	Per unit increase	*0.99*	*(0.98, 0.99)*	0.99	(0.98, 1.004)	0.99	(0.98, 1.004)	*0.98*	*(0.96, 0.99)*
HADS anxiety	Per unit increase	*0.93*	*(0.88, 0.97)*	*0.95*	*(0.90, 1.01)*	*0.95*	*(0.90, 1.01)*	*0.87*	*(0.81, 0.93)*
HADS depression	Per unit increase	*0.91*	*(0.86, 0.96)*	*0.94*	*(0.89, 1.001)*	*0.95*	*(0.89, 1.004)*	*0.86*	*(0.80, 0.93)*
Spinal VAS	Per unit increase	0.97	(0.88, 1.06)	1.02	(0.92, 1.13)	*1.11*	*(0.99, 1.24)*	*0.84*	*(0.74, 0.96)*

*Note: italics* indicate variable for stepwise model (*P* < 0.2).

OR: odds ratio; ref: reference category; ASAS: assessment in ankylosing spondylitis; SF-12 MCS: short form 12 mental component score; SF-12 PCS: short form 12 physical component score; BASDAI: Bath ankylosing spondylitis disease activity index; BASFI: Bath ankylosing spondylitis functional index; ASDAS: ankylosing spondylitis disease activity score; BASG: Bath ankylosing spondylitis global score; Bath ankylosing spondylitis metrology index; ASQoL: ankylosing spondylitis quality of life index; HADS: hospital anxiety and depression scale; VAS: visual analogue scale.

**Table 6:** Baseline factors associated with response at follow-up (stepwise logistic regression models)

		ASAS 20 response		ASAS 40 response		ASDAS ≥1.1 reduction		ASDAS score <2.1	
		(model *n* = 261)		(model *n *= 326)		(model *n* = 253)		(model *n* = 239)	
Participants meeting response criteria (%)		52%		33%		47%		35%	
Baseline variables		OR	(95% CI)	OR	(95% CI)	OR	(95% CI)	OR	(95% CI)
Employment	Full-time	Ref	–	Ref	–	Ref	–	Ref	–
	Part-time	0.48	(0.23, 1.03)	0.38	(0.17, 0.84)	0.39	(0.15, 1.02)	0.28	(0.11, 0.74)
	Unpaid/seeking	1.80	(0.53, 6.13)	1.16	(0.38, 3.53)	0.23	(0.04, 1.26)	0.24	(0.05, 1.28)
	Retired	0.67	(0.31, 1.47)	1.52	(0.68, 3.43)	1.08	(0.37, 3.15)	0.90	(0.33, 2.47)
	Retired/unemployed due to ill-health	0.37	(0.18, 0.76)	0.26	(0.11, 0.63)	0.29	(0.11, 0.76)	0.04	(0.01, 0.34)
	Student	Low *N*.		0.99	(0.13, 7.73)	2.14	(0.12, 38.22)	2.69	(0.26, 27.50)
BMI	Per unit increase	0.96	(0.91, 1.003)						
Education	Secondary school			Ref	–			Ref	–
	Apprenticeship			1.99	(0.85, 4.64)			1.43	(0.50, 4.08)
	College			1.41	(0.72, 2.75)			1.01	(0.43, 2.36)
	University degree			2.82	(1.41, 5.61)			1.72	(0.72, 4.10)
	Further degree			1.27	(0.47, 3.41)			2.62	(0.83, 8.28)
Enthesitis	Yes *vs* no					3.85	(1.33, 11.11)		
SF-12-MCS	Per unit increase					1.05	(1.02, 1.09)	1.05	(1.01, 1.08)
ASDAS	Per unit increase					4.43	(2.83, 6.94)		
Gender	Female *vs* male					0.59	(0.29, 1.20)		
Comorbidity count	Per unit increase			0.64	(0.45, 0.92)	0.57	(0.37, 0.88)	0.60	(0.38, 0.95)
HADs anxiety	Per unit increase	0.94	(0.88, 0.99)						
Model fit	ROC area under curve (95% CI)	0.68 (0.61, 0.74)		0.71 (0.65, 0.77)		0.85 (0.80, 0.89)		0.81 (0.75, 0.86)	
	Sensitivity/Specificity (%)	70.7/56.3		32.1/88.5		73.3/78.9		56.5/81.8	
	PPV/NPV	62.7/64.9		58.3/72.2		75.9/76.6		63.2/77.3	

OR: odds ratio; ref: reference category; ASAS: assessment in ankylosing spondylitis; ASDAS: ankylosing spondylitis disease activity score; SF-12 MCS: short form 12 mental component score; SF-12 PCS: short form 12 physical component score; HADS: hospital anxiety and depression scale; ROC: receiver operating characteristic; PPV: positive predictive value; NPV: negative predictive value.

### Predictors of meeting ASAS40 response

At follow-up, 33% (*n* = 111) of patients met ASAS40 response criteria. As with ASAS20, in the final model, not being in full-time employment was associated with lack of response. Additional factors included education, in which the less educated were less likely to respond and a greater number of comorbidities [OR of response 0.64 per additional comorbidity 95% CI (0.45, 0.92)]. In contrast to the ASAS20 model, BMI and anxiety were not included in the final ASAS40 model (although both were initially associated with ASAS40 outcome). This model demonstrated a similar level of fit to the ASAS20 model (ROC curve 0.71) with PPV and NPV of 58% and 72% respectively.

### Predictors of meeting a clinically important reduction in ASDAS score

Of 261 participants, 122 (47%) met criteria for a clinically important reduction in ASDAS, and as with the ASAS20/40 models, there were a wide variety of (mostly similar) variables that predicted lack of response. Six were included as independent predictors in the multivariable analysis (Table 6). As in the ASAS response models, not being in full-time employment was included in the multivariable model. Additional factors included female gender, lower baseline ASDAS score [4.43 per unit increase 95% CI (2.83, 6.94)], poorer mental health [SF-12 MCS: 1.05 per unit increase 95% CI (1.02, 1.09)], more comorbidities [0.57 per additional comorbidity 95% CI (0.37, 0.88)] and the absence of enthesitis [3.85 95% CI (1.33, 11.11)]. The final model demonstrated a high level of fit (ROC 0.85, with PPV 76%, and NPV 77%).

### Predictors of moving to a moderate or inactive (ASDAS) disease state

There were 249 patients eligible for this analysis (i.e. who had high/very high disease activity at baseline) and who provided follow-up data. At follow-up, 87 (35%) were classified as having moderate or inactive disease. The factors associated with lack of response on univariable analysis were wide-ranging and very similar to those which predicted ASAS response although higher initial ASDAS disease activity was associated with lower likelihood of achieving a moderate or inactive disease state. Four factors were included as independent predictors of lack of response at follow-up (Table 6). Not being in full-time employment and lower education level were associated with lack of response, as was poorer mental health [SF-12 MCS: 1.05 per unit increase 95% CI (1.01, 1.08)] and more comorbidities [0.60 per additional comorbidity 95% CI (0.38, 0.95)]. The final model demonstrated a good level of fit (ROC 0.81) with PPV 63% and NPV 77%.

Running the multivariable models above and adding data on the total score from the 2011 fibromyalgia criteria, in the subset of subjects with this data available (*n* = 141 in whom at least one response criteria could be calculated), showed that it did not result in important improvement in fit to any of the models (data not shown).

## Discussion

Irrespective of the specific axSpA response criteria used, adverse socio-economic factors and fewer years of education predicted poorer response to initial anti-TNFα therapy, as did not working full-time. Clinical factors (co-morbidities) and patient-reported factors (poor mental health) were also associated with lack of response. With the exception of the model predicting ASDAS reduction, no axSpA-specific clinical variables were independently predictive of poor outcome and neither were lifestyle factors (smoking and alcohol consumption). The performance of the models was good, and in particular there was high NPV for the ASDAS models (77%) indicating the ability to predict well those unlikely to meet response criteria. Studies that have reported that axSpA-specific factors predict response to TNFα have generally not collected information on socio-economic factors and may therefore have been affected by unmeasured confounding [e.g. 31, 32]

The patients in the register were recruited from more than eighty centres throughout the Great Britain and as such represent a ‘real-world’ use of anti-TNFα with greater heterogeneity of clinical features than are present in trial populations. However, in contrast, it was necessary to be flexible with respect to timing of assessing outcome in this study as, although patients were scheduled to be followed up at 3 and 6 months after commencing their first anti-TNFα therapy, the follow-up visit did not always happen at these times. A follow-up of 3–4 months reflects UK clinical practice. Indeed the National Institute of Health and Clinical Excellence (NICE) recommends follow-up at 12 weeks and that anti-TNF therapy should only be continued if there is ‘clear evidence of response’ at this point [33]. With the knowledge that there may be some delays to follow-up in the real world, we have chosen a longer window for first follow-up to occur. The first visit in a time window rather than at a specific time point has been used. This option was chosen because patients who attend a scheduled appointment may be more likely to have clinical issues such as lack of efficacy or an adverse event in comparison to those who choose not to attend. However, if a patient had experienced a serious adverse event necessitating hospital admission this would also result in failure to attend an appointment. Thus, using a wider window of follow-up increased the numbers whose outcome was assessed and potentially decreased any ‘non-participation’ bias. In reality, the distribution of follow-up time shows that half the participants were seen within a 5-week window. Secondly, the patient-reported and clinical outcomes were not collected at the same time. The follow-up study questionnaire was issued at the time follow-up was due and therefore there was a difference between this and the time the actual clinic visit took place. The median difference between the follow-up visit and follow-up questionnaire was 13 days (IQR –1, 34). Thirdly, at the time of follow-up, some patients had stopped their anti-TNFα therapy (e.g. due to an adverse event) and thus the interpretation of our results is the prediction of outcome amongst patients who commence their first anti-TNFα rather than outcome while patients are still taking such therapy. However, this is the most relevant question for a clinician facing the decision on whether to commence a patient on a specific therapy: ‘*If I choose to prescribe anti TNFα for this patient, how likely is it that they will/will not have achieved a positive response in around 3–4 months?’*.

There have only been few studies examining response to biologic therapy in patients with axSpA. Molto *et al.* hypothesized that patients with high enthesitis and/or disease activity scores (BASDAI) may be less likely to respond (on the basis that this could indicate co-morbid fibromyalgia), but found that neither disease index influenced likelihood of meeting ASAS40 or ASAS partial remission criteria at 12 weeks [34]. Callhoff *et al.* reported that the efficacy of anti-TNFα therapy was not related to specific criteria satisfied (i.e. AS or non-radiographic axial spondyloarthritis) [35]. The results of the current study generally support these conclusions although the presence of enthesitis and disease activity (as measured by ASDAS) were the sole disease-related factors found to relate to response in this study, and only for a single outcome measure (reduction in ASDAS). An Italian multicentre retrospective study of ∼300 patients found that the presence of enthesitis and psoriasis was associated with lower likelihood of patients achieving at least partial remission (which was defined as <20 mm in the four domains of global assessment, spinal pain, function and intensity/duration of morning stiffness). This study, however, did not collect any information on socio-economic factors [36]. A report that appears to be from the same study examines response by gender and found that females were less likely to achieve partial remission than males. It states that this was true also in a multivariable model, but does not indicate what was included in the model and there was no mention of any socio-economic factors collected [37]. In the current study, for three out of the four response criteria females were less likely to respond, although none were statistically significant and adding gender did not improve the fit of any of the multivariable models.

With respect to lifestyle factors predicting response, a recent systematic review and meta-analysis has quantified the effect of obesity on response to anti TNFα therapy across a range of immune-mediated inflammatory diseases. Within this study, a sub-analysis of six studies of spondyloarthropathies (including 966 patients of whom 14% were obese) found increased odds of non-response to therapy, but with considerable uncertainty OR 3.4 95% CI (1.3, 8.5) [38]. In the current study, higher levels of BMI were associated with non-response (the OR per unit increase varied between 0.94 and 0.97 depending on the response criteria). However, only in the multivariable model for ASAS20 did it significantly improve model fit. The role of smoking in treatment response is less clear. Current smoking has been related to higher disease activity in patients with axSpA and AS [39, 40], including observations of a pack-year/disease activity dose-response [41]. Although studies of patients with axSpA included in the Swiss Clinical Quality Management Cohort as well as those in the DANBIO Danish nationwide registry found that smokers had odds of around 0.5 in meeting BASDAI50 response criteria (in comparison to non-smokers) [42, 43] data from the BSRBR-AS did not find smoking to be a predictor of response to TNFα inhibitors and suggested that previous observations of an association may be explained by methodological factors [44].

What has this study added to our existing knowledge? It has demonstrated that, generally, disease specific factors do not predict response to first anti-TNFα therapy. The factors that predict response across different criteria are not modifiable at least by the rheumatologist in the clinic. Factors such as level of deprivation, level of education and employment status are, however, identifying persons who may need additional management in order to derive the benefit that other patients receive from anti-TNFα therapy. This may include support for self-management including education, or non-pharmacological therapy (such as physical therapy or input from an occupational therapist), and this could usefully be the focus for testing in future studies. Poor mental health is also a marker of lower likelihood of response across criteria and patients with such markers of poor response may need specific assessment and behavioural therapy or psychological support in order for to derive benefit from pharmacological therapy. Retiring or not being in employment due to ill-health is a strong and consistent predictor of non-response across the models; future work should identify the reasons (disease-specific or contextual) why this is the case.

In conclusion, the statistical models in this study identify patients with a high likelihood (70–80%) of not responding to their first biologic therapy for axSpA—some (such as mental health) are modifiable, whereas others (such as social and economic factors) do not lend themselves to modification in the clinic—but identify patients who otherwise are unlikely to benefit from biologic therapy alone. Priority should be focused on how we ensure that these patients receive the benefits that many patients derive from such therapies. It also emphasizes that examination of predictors of non-response to pharmacologic therapy in inflammatory arthritis must consider the importance of socio-economic factors.
